# Jamaica’s Critically Endangered Butterfly: A Review of the Biology and Conservation Status of the Homerus Swallowtail (*Papilio* (*Pterourus*) *homerus* Fabricius)

**DOI:** 10.3390/insects8030068

**Published:** 2017-07-10

**Authors:** Matthew S. Lehnert, Valerie R. Kramer, John E. Rawlins, Vanessa Verdecia, Jaret C. Daniels

**Affiliations:** 1 Department of Biological Sciences, Kent State University at Stark, North Canton, OH 44720, USA; vkramer@kent.edu; 2 Carnegie Museum of Natural History, Pittsburgh, PA 15213, USA; rawlinsj@carnegiemnh.org (J.E.R.); verdeciav@carnegiemnh.org (V.V.); 3 McGuire Center for Lepidoptera and Biodiversity, Florida Museum of Natural History, Gainesville, FL 32611, USA; jdaniels@flmnh.ufl.edu; 4 Department of Entomology and Nematology, University of Florida, Gainesville, FL 32611, USA

**Keywords:** Homerus swallowtail, Jamaica, *Papilio homerus*, conservation, endangered species, flagship species

## Abstract

The Homerus swallowtail, *Papilio* (*Pterourus*) *homerus* Fabricius, is listed as an endangered species and is endemic to the Caribbean island of Jamaica. The largest butterfly in the Western Hemisphere, *P. homerus* once inhabited seven of Jamaica’s 14 parishes and consisted of at least three populations; however, now only two stronghold populations remain, a western population in the rugged Cockpit Country and an eastern population in the Blue and John Crow Mountains. Despite numerous studies of its life history, much about the population biology, including estimates of total numbers of individuals in each population, remains unknown. In addition, a breeding program is needed to establish an experimental population, which could be used to augment wild populations and ensure the continued survival of the species. Here, we present a review of the biology of *P. homerus* and recommendations for a conservation plan.

## 1. Introduction

**Figure 1:**
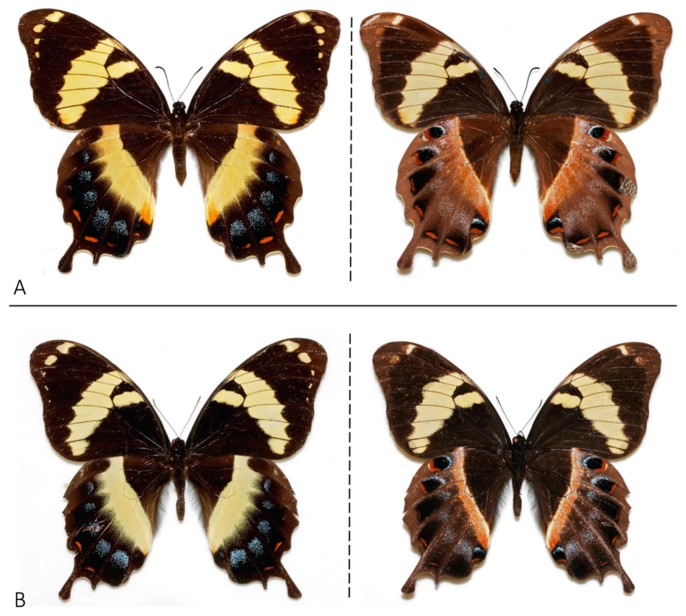
Photographs of representative *Papilio homerus* from the eastern population. (**A**) Female of *P. homerus* (dorsal on left, ventral on right) collected from the Blue Mountains, Jamaica (1959). (**B**) Male of *P. homerus* collected at Corn Puss Gap, Jamaica (1986). All photographs are used with permission from Warren et al. (2016) and acquired from http://butterfliesofamerica.com/L/t/Pterourus_homerus_a.htm.

**Figure 2:**
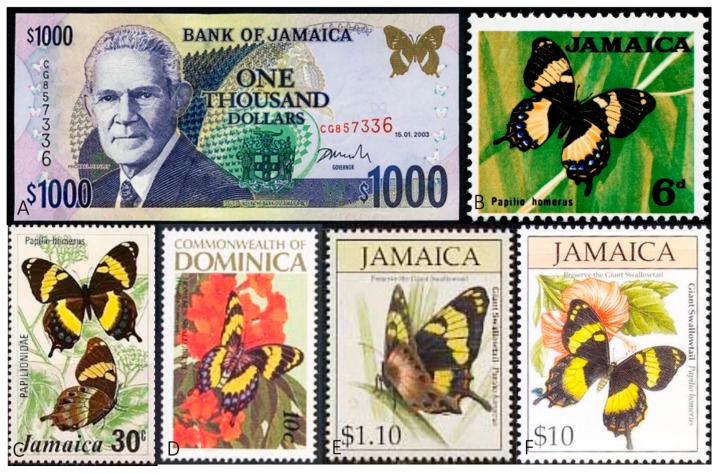
Printings and postage stamps of *Papilio homerus* that contribute to its status as a flagship species. (**A**) The Jamaican $1000 bill features *P. homerus* in the upper-right corner. Representative stamps issued in 1964 (**B**), 1975 (**C**), 1989 (**D**), and 1994 (**E**,**F**) that feature *P. homerus*.

The Homerus swallowtail (*Papilio* (*Pterourus*) *homerus* Fabricius 1793) (Figure 1) is the largest butterfly in the Western Hemisphere [1,2] and is critically endangered [3,4,5]. Endemic to the island of Jamaica [6,7], *P. homerus* is a flagship species that symbolizes the need for conservation efforts [8] and has been featured on postage stamps and the Jamaican $1000 bill (Figure 2). *Papilio homerus* is listed in the IUCN Red Data Book, *Threatened Swallowtail Butterflies of the World* [3], and is protected as an Appendix I species by the Convention for International Trade in Endangered Species (CITES) and the Jamaican Wildlife Act of 1988. The range of this enigmatic butterfly, possibly less than 10 km^2^ for each of the two remaining populations, continues to dwindle, particularly due to habitat destruction [4] and recent mining initiatives [7,9]. In addition, the extant populations of *P. homerus* reside in areas of Jamaica famous for their difficult terrain [5,7,10,11], which further confounds the ability to perform the long-term field studies necessary for understanding the biology and population dynamics for this species. Despite these complications, lab and field studies have given insight into the ecology, life history, and behavior of *P. homerus*. Here, we provide an overview of the biology of *P. homerus*, including knowledge gaps and objectives for future conservation efforts for this imperiled butterfly.

## 2. *Papilio homerus* Historical Records

**Figure 3:**
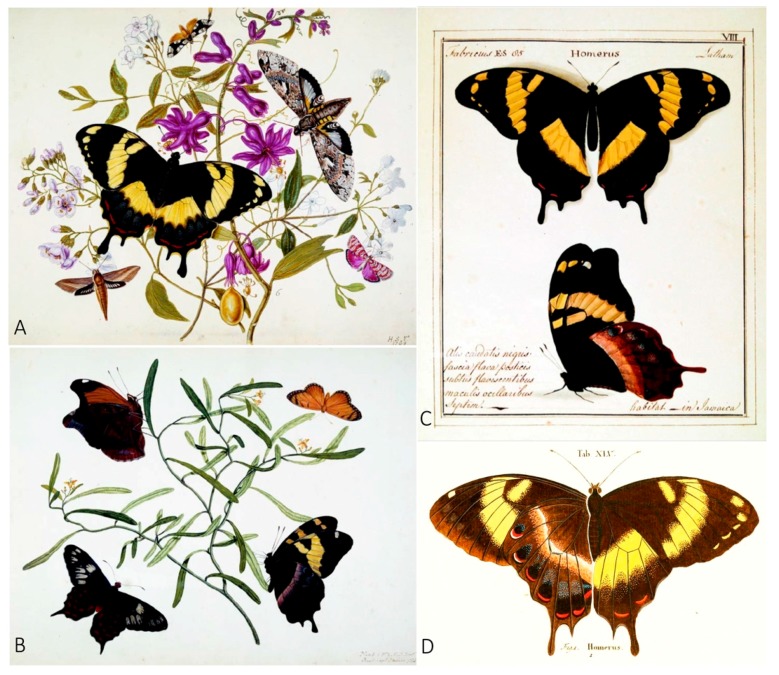
Paintings of *Papilio homerus* from the 18th century. Putative first two paintings of *P. homerus* by Henry Seymer (1768, (**A**)) and with his son, Henry Junior (1773, (**B**)). (**C**) Painting of *P. homerus* by William Jones (1783–1785), which was used by Fabricius in 1793 to describe the species [12]. (**D**) Painting of *P. homerus* by Esper, Eugen Johann Christoph (1796). Images (**A**) and (**B**) are shown with permission from R.I. Vane-Wright. Image (**C**) is shown with permission from Oxford University Museum of Natural History, and image (**D**) shown with permission by Warren et al. (2016) and acquired from http://butterfliesofamerica.com/L/t/Pterourus_homerus_a.htm.

The grandeur of the Homerus swallowtail has inspired artists since its discovery by Western culture. *Papilio homerus* was first described by Johan Christian Fabricius [12] (not from a specimen, but from a painting by William Jones, Esq. of Chelsea, Figure 3C) [13], who, awestruck by its beauty, named the butterfly *Papilio homerus* in honor of the Greek poet, Homer, the author of *The Iliad* and *The Odyssey*. Even before Jones’ paintings, *P. homerus* was the subject of paintings by Henry Seymer (1768, Figure 3A) and collaborative paintings with his son, Henry Seymer Jr. (1773, Figure 3B) [13]. Three years after the description by Fabricius, the German entomologist Eugenius Johann Christoph Esper illustrated *P. homerus* (Figure 3D) in his series of booklets, *Die Schmetterlinge in Abbildungen nach der Natur mit Beschreibungen* [14]. Much of the biology of *P. homerus*, however, remained unreported until Rutherford [15] and Gosse [16] provided information of the immature stages and general habits of the butterfly. The morphology of the immature stages of *P. homerus* was also discussed by Panton [17] and Taylor [18]. Many of these early reports were anecdotal and it was not until descriptions by André Avinoff and his nephew, Nicholas Shoumatoff, that details were provided about the biology and preferred habitats of the adult life stage ([19], discussed in [20]). Despite recent efforts to study the biology of *P. homerus* [4,5,6,7,10,11,21,22,23,24], there are still gaps in our knowledge about the species, particularly about the biology of the adults, the total number of individuals remaining in the stronghold populations, and population dynamics. In addition, information about the genetic architecture of *P. homerus* populations is completely lacking because no genetic studies have been performed, which is necessary for the development of an effective conservation plan.

## 3. Morphology and Life History Traits

**Figure 4:**
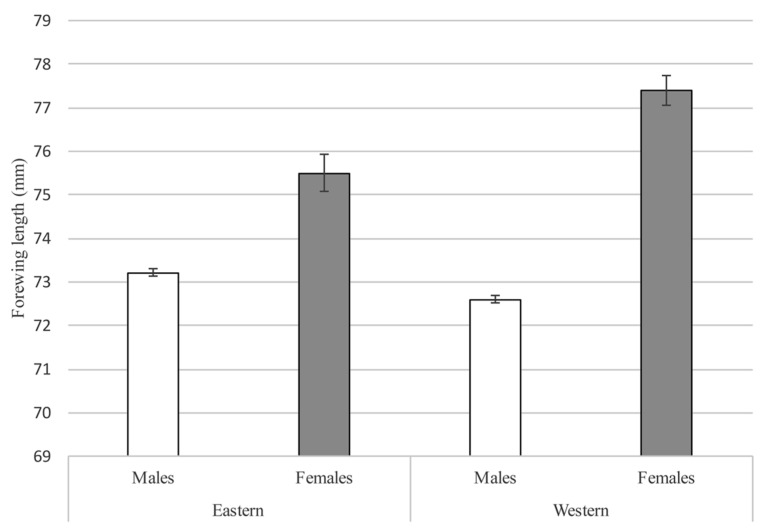
Forewing length measurements (mean ± SE) of *Papilio homerus* collected from the eastern and western populations. Females of *P. homerus* from the eastern population (*n* = 2) had significantly larger forewing lengths than males (*n* = 19) (*p* = 0.03). A similar pattern is shown with *P. homerus* collected from the western population, where females (*n* = 5) had significantly larger forewing lengths than males (*n* = 15) (*p* = 0.02). In general, females are significantly larger than males (*n* = 8, 34, respectively; *p* = 0.04), similar to previous reports [4,6,7]. There were no significant differences in forewing length measurements in males or females between populations (*p* ≥ 0.05). All individuals measured from the eastern population and two males from the western population were acquired from the Carnegie Museum of Natural History (Pittsburgh, PA) collection. The remaining individuals measured from the western population are reported by Lehnert (2008). A two-sample unequal variance t-test was used to test for significant differences (*p* < 0.05). Although no significant differences were found within sexes between populations, we suggest a future and more rigorous morphometric analysis is needed to compare populations, which would include a larger sample size, additional wing measurements, and details about the genitalia.

Morphological studies of *P. homerus* have a diverse history [6,10,17,18,23], but many of these are descriptions pertaining solely to individuals from the eastern population. No previous investigations have provided details about the morphology of individuals from the western population, and considering the geographic distance, a future comparative morphometric study might reveal evidence of isolation between the extant populations. Here, we provide a brief outline of the morphology of the life stages and a comparison of the adult forewing lengths, an indicator of size [25], among extant populations as potential evidence of morphological trait differentiation (Figure 4). 

### 3.1. Eggs

Eggs (ova) of *P. homerus* are smooth, nearly spherical, and approximately 1.5–2.0 mm in diameter. Following oviposition, the eggs are pale green, but change to yellow and then dark brown before eclosion [6,7,10].

### 3.2. Larval Stages

Larvae of *P. homerus* go through a transition in coloration where the early instars resemble bird droppings and later, larger instars are often green, which is considered to have adaptive value as an anti-predation mechanism [22,26]. Here, we provide a summarized report using descriptions provided by Panton [17], Turner [10], and Emmel and Garraway [6]. The first and second instars of *P. homerus* are generally dark brown with abdominal segments that are white. The first instar has numerous setae and attains a length of approximately 9 mm. The duration of the first instar is five days. In the second instar, the setae are reduced and the thoracic region is enlarged with a hump-like appearance. The second instar attains a length of approximately 15 mm and lasts five days. The third instar has a white dorsal saddle and prominent black and white eyespots on the third thoracic segment. The third instar lasts nine days and the larvae attain a length of approximately 26 mm. The fourth and fifth instars are similar in color and overall appearance. After molting, the fourth instar appears similar to the third instar, but then transitions to a primarily green color. The thoracic segments are swollen and the metathoracic segment has a pair of blue and yellow eyespots connected with a brown band, giving the larvae the appearance of a small snake or lizard. The fourth instar lasts ten days and attains a length of 40 mm. The fifth instar lasts approximately 20 days and the larvae reach a length of 70 mm. Similar to other larval Papilionidae, the larvae of *P. homerus* have osmeteria [22], which are forked scent glands that can be everted from a slit located in the dorsal prothoracic region behind the head. The osmeteria of the first three instars are pink, but are red in the last two instars [22].

### 3.3. Pre-pupae

After approximately 45–50 days, the color of the larvae changes to dark gray. Larvae spin a cremasteral pad and thoracic girdle, then pupate 48 hours later.

### 3.4. Pupae

The pupae (chrysalides) occur in several color forms, including brown, gray, and mixed brown with olive-green. There are six white dorsal spots and two lateral spots (one on each side). The larvae pupate on stems and branches of *Hernandia* plants and other plant species [10]. 

### 3.5. Adults

*Papilio homerus* is the largest species in the genus with a forewing length that averages 75 mm [5,6], and some reported female specimens have a forewing length of 90 mm [27]. The sexes of *P. homerus* are similar in coloration and pattern and the females are larger in size [5,6] (Figure 4). The dorsal surface of the forewing and hindwing has a base color of dark brown to blackish with a broad, yellow discal band extending across both wings. The hindwing has powdery-blue postdiscal spots and brick-red submarginal lunules and large spatulate tails (Figure 1). The ventral surface of the wings also has a dark brown-black base color, but the yellow discal band is narrower with sporadic blue scales [6,10].

## 4. *Papilio homerus* Biology 

### 4.1. Host Plants and Behavior of Larvae

The only confirmed host plants for *P. homerus* are *Hernandia jamaicensis* Britton and Harris (Hernandiaceae) (locally known as water mahoe and water wood) in the western population and *H. catalpaefolia* Britton and Harris (pumpkin wood and suck axe) in the eastern population [6,10]. Neither extant population of *P. homerus* has access to both *Hernandia* species; *H. jamaicensis* is restricted to the western and northwestern parishes, whereas *H. catalpaefolia* is found only in the eastern parishes, such as Portland and St. Thomas [5]. Both species of *Hernandia* are endemic to Jamaica, common in *P. homerus* habitat, and found near streams and ravines [4,6]. Females of *P. homerus* have been observed to oviposit on a related *Ocotea* sp., probably *O. leucoxylon* (Sw.) Gómez Maza (Lauraceae) (loblolly sweetwood), in the Cockpit Country [28] and at Corn Puss Gap [10], which suggests that this plant species might serve as a host plant, but this requires further study. *Ocotea* trees also occur on Mount Diablo and might have been the primary host plant for the former central population of *P. homerus*. Another potential host plant at Mount Diablo is *H. senora* L., which was introduced from Mexico, but is grown only in cultivation [5,6,7]. 

Larvae are not easily disturbed, at least by human presence. The osmeteria, for instance, were initially reported as being absent [17,18,29], a unique character among the swallowtail butterflies. Nearly 100 years after the original descriptions of the larval stages, Turner [10] reported the presence of osmeteria, which were later described by Garraway and Parnell [22]. Larvae need to be thoroughly provoked in order to extrude osmeteria [22], which is likely why it was originally reported as being absent. 

Adult *P. homerus* reared as larvae in less than 100% humidity fail to properly expand their wings upon emergence from the chrysalis [7]. High humidity is apparently an essential component for *P. homerus* larvae [7,30], which have been observed to drink droplets of water from leaves of *H. catalpaefolia* [6], and is likely a factor the influences habitat availability. The larvae are primarily nocturnal feeders and spend the day on the top of a *H. catalpaefolia* leaf where they construct a silken retreat [6]. Larvae have been recorded most months of the year in the eastern population [23]. There are no intensive studies of the immature stages from the western population.

### 4.2. Adult Population Size Estimates and Behavior

Estimates of the remaining number of *P. homerus* in the extant metapopulations are needed. The difficult terrain coupled with the complex subpopulation structure have made population size estimates difficult, and many of these subpopulations show fluctuations in numbers and perhaps extinctions [7]. These putative extinctions could be seasonal movements to other localities or attributed to the difficulty of finding specimens when numbers are low. Although there are numerous reported sightings of *P. homerus*, e.g., 103 adults observed by Turner [10] in both populations, 200 adults observed flying between 1981 and 1986 in the eastern population [23], and over 200 adults observed at Fishbrook (near Millbank) between 1991 and 1993 [7], each study period might consist of observations of the same individuals and the number of *P. homerus* is likely fewer. 

There have been two noteworthy attempts to estimate population sizes using Mark-Release-Recapture (MRR) methods [5,23]. In April 1991, 20 males and five females of *P. homerus* were captured from the eastern population and the only recapture was of an individual previously captured 30 min prior [7]. The second MRR study estimated the size of a subpopulation of *P. homerus* near the town of Niagara in the Cockpit Country in July–August 2004 [5]. This study resulted in the capture of 18 individuals and 17 recaptures (estimated size of subpopulation: fewer than 50 individuals); however, many of the recaptures were of the same specimen (only six different individuals were recaptured) [5]. No *P. homerus* were observed in a follow-up study in the western population during the winter months (December 2004–January 2005) [5], but seasonal fluctuations in numbers of adult *P. homerus* are known [7]. 

Sampling wild populations has indicated a male-biased sex ratio at 3.5:1 in the eastern population (*n* = 27) [23] and 2.6:1in the western population (*n* = 18) [4]. These sex ratios are probably skewed because of the bias in sampling methods coupled with sex specific behavioral differences. Lehnert [4], for instance, used a clearing in the rainforest with a pathway to collect *P. homerus*, which might have consisted of ideal habitat for patrolling males. The sex ratio is likely closer to 1:1, as indicated in captive rearing experiments (*n* = 19) [7]. 

Most sightings of *P. homerus* occur between 9 a.m. and 2 p.m. [5,7], but activity can begin as early as 8 a.m. and continue until 6 p.m. [5,19]. From 9–10 a.m., *P. homerus* of both sexes can be observed basking in sunlight on the tops of leaves at high elevations in the canopy and on lower shrubs (for periods sometimes greater than 30 min) or feeding on various nectar sources [5,6,7]. Observations of feeding behaviors of both sexes suggest that the adults are opportunistic flower visitors and feed from flowers of *Lantana camara* (Verbenaceae), *H. catalpaefolia*, *Hibiscus rosasinensis* (Malvaceae), *Urena lobata* (Malvaceae), *Entada gigas* (Fabaceae), *Psophocarpus palustris* (Papilionaceae), *Cissus* sp. (Vitaceae), *Mecranium* sp. (Melastomataceae), *Hedychium coronarium* (Zingiberaceae), *Asclepias* sp. (probably *A. curassavica*) (Asclepiadaceae), *Pachystachys coccinea* (Acanthaceae), *Tabernaemontana ochroleuca* (Apocynaceae), *Spathodea campanulata* (Bignoniaceae), and *Bidens* sp. (probably *B. pilosa*) (Compositae) [5,7,10,31]. Gross observations of the proboscis of *P. homerus* indicate it is similar in structural architecture as other examined *Papilio* spp. [32,33] and has a proboscis adapted for flower feeding [34], but this requires further study. There are no reports of *P. homerus* feeding on food sources other than nectar or of males puddling on wetted soil [35,36].

Males of *P. homerus* patrol territories and engage in territorial encounters with other male *P. homerus* [5,7,24]. Territories are topographically defined and consist of forest clearings where nectar sources are common [7,24], characteristics attractive to other territorial butterfly species [37]. As described by Wickman and Wiklund [38], Lepidoptera that engage in disputes over a territory display three types of behaviors, a spinning-wheel flight, a horizontal flight-pursuit, and a horizontal-spiraling flight-pursuit. Males of *P. homerus* display behaviors from all three categories, but the horizontal-spiraling flight-pursuit has been the most common observed [24]. 

Territorial encounters began when an intruding male *P. homerus* entered the territory of a resident male. Both butterflies would fly towards each other and collide, resulting in an impact that was audible [24] from at least 3 m distance (M.S. Lehnert, personal observation). The collision was followed by a spinning-wheel flight where both males rapidly flew circles around each other until one male chased the other male out of the territory. The winner, usually the original resident [39], would return to the territory in approximately 3 min [24]. Given the sound of the initial impact between males and the continued audible engagement (wings hitting one another during spinning-wheel flights), it was hypothesized that territorial encounters produced the extensive wing damage observed on some territorial males; approximately 93% of a hind wing was missing from one individual that occupied a territory over a period of 19 days [24,40]. Conspecific intersexual interactions for *P. homerus* are not well documented. Courtship behavior, for instance, is unknown and there is only one documented case of observed copulation in the eastern population in 1991 [23]. 

## 5. Phylogeny and Biogeography

**Figure 5:**
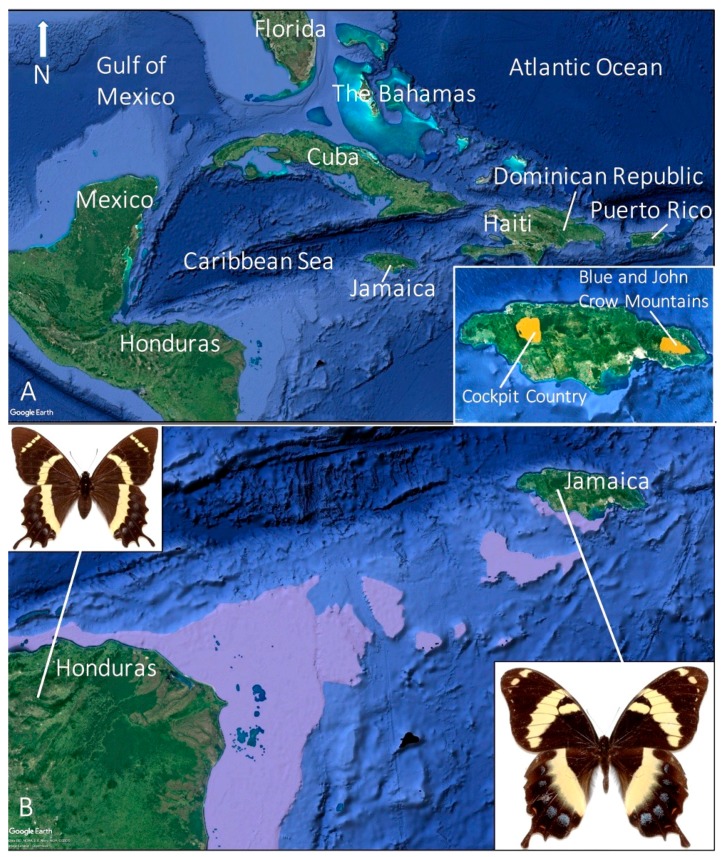
Maps of Central America and the Caribbean showing the distribution of *Papilio homerus*. (**A**) Map showing the location of Jamaica relative to the other Caribbean islands and Central America. The inset (bottom right) shows the range of the extant *P. homerus* populations (yellow) in the Cockpit Country and the Blue and John Crow Mountains, Jamaica. (**B**) *Papilio garamas* (from Honduras and other regions of Central America, top left image) and *P. homerus* (only found in Jamaica, bottom right) putatively share a recent common ancestor. Fluctuations in the sea level might have extended the mainland of Central America and created islands in the past (areas shown in purple) that would have acted as stepping stones for the common ancestors of *P. garamas* and *P. homerus* to move to Jamaica and speciate. Shown here is a female of *P. garamas electryon* Bates 1864 captured from El Salvador in 1969. All maps were acquired from Google Earth^®^.

Morphological and molecular analyses have provided evidence for the evolutionary relationships among the swallowtail butterflies (Papilionidae) [41,42,43,44]. The genus *Papilio* is currently accepted as being comprised of four groups, including the *machaon*-clade (143 species), the *Heraclides*-clade (29 species), a grouping that contains one species, *P. alexanor* Esper 1799, and the *Pterourus*-clade (33 species) [44], which includes *P. homerus*. Morphological analyses suggest that *P. garamas* Geyer (Figure 5), shares a recent common ancestor with *P. homerus* [6]: *P. garamas* is a smaller, Lauraceae-feeding species from Central America with similar markings as *P. homerus* [45]. 

Jamaica lies approximately 150 km south of Cuba and 610 km northeast of Honduras; however, there have been many instances in the past (e.g., 10 mya, 6 mya, 20–10,000 years ago) when sea levels were lower and the mainland of Central America was at least 300 km closer to Jamaica [46] (Figure 5). During periods of low sea levels, other small islands might have been present between the mainland and Jamaica creating a series of land masses for butterflies, such as the ancestor of *P. homerus* and *P. garamas*, to spread and speciate from mainland populations (Figure 5B). 

Jamaica is the third largest Caribbean island (11,740 km^2^) and has topographical features that are primarily high in elevation, particularly in the eastern part of the island, where the Blue and John Crow Mountain ranges are found (maximum elevation of 2155 m). The Central Upland Plateau characterizes the middle of the island. A large portion of the western side of the island has rolling mountains that are interspersed by deep valleys and sinkholes—an area referred to as the “Cockpit Country”—because it reminded early settlers of the pits used for cockfighting [6]. 

## 6. Past and Present Distributions and Habitat Preference

Based on reported sightings and collected individuals, *P. homerus* inhabited seven of Jamaica’s 14 parishes and were clustered into three groupings: the central, eastern, and western populations (Figure 5A) [5]. The central population was located near Mt. Diablo, the eastern population is located at the junction of the John Crow and Blue Mountain ranges, and the western population is in the Cockpit Country [4]. The interpopulation dynamics are unknown and individuals of *P. homerus* have not been collected between these populations, which suggests that the central population was a true population, i.e., individuals collected there were not strays from the peripheral populations.

### 6.1. Central Population

Of the three known populations of *P. homerus*, the central population remains the least understood and has likely disappeared [7,10]. The region is characterized as an upland plateau that ranges from 600 to 1000 m in altitude [7] with sightings of *P. homerus* primarily restricted to the vicinity of Mt. Diablo [6,31], which is approximately 70 km from the eastern and western populations. Despite numerous scouting trips to the region in the 1930s [19] and in 2006–2007 [7], no *P. homerus* have been sighted from the central population since 1925 [47]. Logging and the expansion of agriculture resulted in extensive habitat destruction through the center of Jamaica earlier than in other areas inhabited by *P. homerus* [6], which might have resulted in the extinction of this population [7,10]. The mating of individuals in the central population might have historically served as a gateway for the sharing of genes between the peripheral populations, therefore preventing genetic bottlenecking. 

The current climate conditions and habitat of the Mt. Diablo region do not make it a potential location for the future release of captive-bred individuals, if a breeding program is established. Although there are remnant forests here that look promising for *P. homerus* populations, the region is drier (annual rainfall of 2000 mm) than the eastern and western populations, which might be unsuitable for *P. homerus* larvae that require high humidity [6,10]. Also, the area lacks either of the native host plants [7], *H. catalpaefolia* and *H. jamaicensis*, but does host *H. senora* L., introduced from Mexico, and *Ocotea* sp. (Lauraceae), which has been used for oviposition by wild *P. homerus* in the eastern [10] and western populations [28].

### 6.2. Eastern Population

Most information about the biology of *P. homerus* comes from studies of the eastern population located at the merger of the Blue and John Crow Mountains, near Corn Puss Gap (610–715 m in altitude), the Rio Grande Valley, and Bath [6,7]. The region obtains a large amount of rainfall due to the location of the mountain ranges along the east trade winds (average annual rainfall at Corn Puss Gap is approximately 7500 mm) [7]. There are numerous villages, such as Millbank, and small subsistence farms in the area. Despite habitat disturbance in the area, the Lower Montane Rainforest regenerates quickly, and the host plant, *H. catalpaefolia*, which is endemic to the region, is common. In general, the topography and environmental conditions of the area represent ideal habitat for *P. homerus* and consist of numerous amphitheatre-like settings with many streams [19]. The establishment of the Blue and John Crow Mountains National Park in 1991, which uses an image of an adult *P. homerus* as its logo, provides additional protection to the region. The large amount of rain, abundance of *H. catalpaefolia*, and amphitheatre-like settings likely contribute to the constancy of this population.

The number of *P. homerus* sightings here, although greater than the other populations, have never been particularly high [6,7]. Records for this population date at least to 1902 when more than 40 specimens were collected over 10 days in July by Robinson [48], and no individuals were seen during the cooler months between December 1906 and March 1907 [49]. Sightings of *P. homerus* were recorded in the region in 1925 [47] and 1939 [19]. Farming and logging increased in the area during the mid-1940s [50], which resulted in habitat loss and *P. homerus* becoming less common; however, farm plots do not last long due to heavy rains and erosion [6], and by the late 1960s *P. homerus* became more abundant [10]. During the early 1980s, as many as 200 adults could be sighted in a single day, although the same individuals were likely sighted multiple times. The same region was explored in August 1986 and only one adult was observed [6]; this exemplifies the fluctuation in the numbers of individuals observed in subpopulations. 

### 6.3. Western Population

The western population of *P. homerus*, first reported by Avinoff and Shoumatoff [19], is located in the Cockpit Country, a 500 km^2^ region where the topography consists of a series of cone-shaped hills and mountains interspersed with deep pits with sinkholes and caves [5,6,7]. There are no easy paths to navigate in the Cockpit Country, making this population less susceptible to illegal collecting and habitat disturbance [7]; however, the lack of accessibility also affects the ability to perform field studies. Marijuana farmers in this region, who have an aversion to strangers trespassing on their property, provide additional protection for this population. The average annual rainfall in the southeastern portion of the Cockpit Country is approximately 2500 mm with the most rainfall occurring in May–June and August–November [6,7]. Although the amount of rainfall here is lower than where the eastern population resides, it is enough to meet the humidity and water requirements for *P. homerus* development [6]. The northern region of the Cockpit Country is drier (1500 mm), which might partially account for the rarity of *P. homerus* in this area [7,11]. In addition to *Ocotea* sp. (used for oviposition) [10], *H. jamaicensis*, an endemic host plant for *P.* homerus, is common in this region [5,11,24]. Sightings of *P. homerus* are primarily restricted to the southeastern portion of the Cockpit Country, near Niagara, Elderslie, Cook’s Bottom, Arcadia, Accompong, and Quickstep [5,6]; however, there have been some recent recordings in northern regions, such as Windsor and Dromily [11].

## 7. Threats to Populations of *Papilio homerus*

### 7.1. Habitat Destruction

Jamaica’s landscape was historically altered at an alarming rate; it was estimated that only 20% of the total forest coverage remained by 1943, and recent deforestation rates were estimated at 0.1% [51]. Although Jamaica is experiencing regrowth in some of its forests, partially due to a decrease in human usage of land, habitat destruction is still of concern. In the 1980s, for instance, the Jamaican government cleared 2000 hectares of rainforest a year to plant fast-growing pine (*Pinus caribbea* var. *honduriensis*) to help Jamaica meet its needs for fuel wood [7]. The pine plantations, which homogenized the landscape, were wiped out by hurricanes [51], resulting in these areas experiencing regrowth of native plant species. In addition, bauxite mining is of concern for the Cockpit Country and the Blue Mountain regions [52]. 

The fragmentation of forests and the production of artificial edges due to clear cutting, agriculture, and commercial building not only threaten the size of *P. homerus* habitat, but also the environmental conditions necessary for *P. homerus* development. Tropical rainforests typically have high humidity, stable temperatures, low light, and little wind due to almost continuous canopy cover [53,54]; however, these conditions are altered when the forest is disturbed or joined with a disturbed landscape. There is an increase in sunlight and temperature in light gaps, a decrease in relative humidity, and increased vulnerability to wind and weather at least 40–60 m into the forest along a newly created edge [54]. Such changes in the microclimate can stress organisms [55,56]. 

### 7.2. Predators and Parasitoids

Parasitism of eggs of *P. homerus* by parasitoids (Hymenoptera), particularly *Ooencyrtus* sp. (Encyrtidae) and *Chrysonotomyta* spp. (Eulophidae), resulted in high mortality [7,21]. There are a number of causes for egg mortality, including predation by insects (e.g., ants) and fungal infections, but 77% of eggs (*n* = 443) collected near Millbank (eastern population) died from parasitoids [21]. Oviposition frequency by *P. homerus* and parasitism were higher in disturbed areas [7]. The trend indicates that parasitoids act in a density-dependent manner with *P. homerus* eggs. Providing disturbed or semi-disturbed areas might be important for *P. homerus* conservation efforts, but the issue of parasitism might require a method of control. 

Pathogenesis is likely the leading cause of death for larvae of *P. homerus*. *Bacillus*, *Clebsiella*, and *Enterobacter* represent the most common genera of bacteria associated with the death of larvae and sometimes pupae [23]. Birds and ants are also recorded predators of larvae [22]. Lizards, e.g., *Anolis* spp., are probably predators of multiple life stages of *P. homerus*. Observations of lizard predation are lacking at this time, but adult *P. homerus* have been captured that exhibit bilateral wing damage in the shape of a lizard bite [57]. 

### 7.3. Poaching

In the 1970s and 1980s, prices for an intact specimen ranged from US $400 to $1500 [6]. Due to increased awareness of the conservation status of *P. homerus* along with new legislation, organized poaching has been reduced mostly to opportunistic collecting [7]. In recent times, local landowners and farmers have acted as a line of defense against poaching, as researchers on insect collecting trips have been confronted numerous times by locals and threatened with police action [7]. Education about conservation biology and raising awareness about the uniqueness and rarity of the species to locals is one of the best lines of defense against illegal collecting.

### 7.4. Climate Change

**Figure 6:**
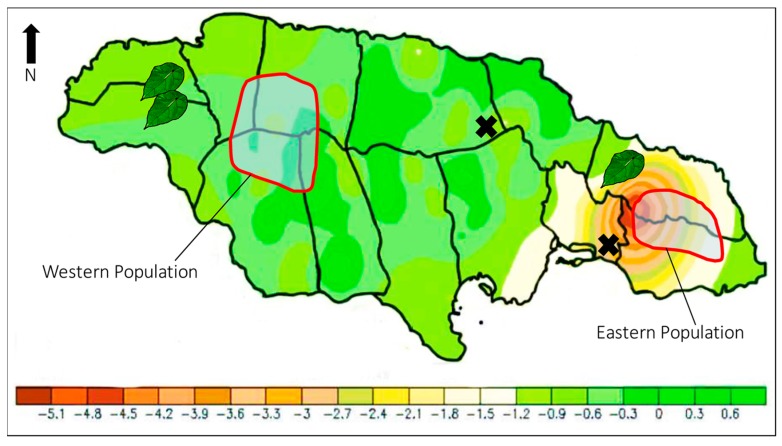
Map showing trend in rainfall in Jamaica from 1992 to 2010. The positive values indicate an increase in rainfall and negative values indicate a decrease (Data source: Meteorological Service of Jamaica). Areas outlined in red indicate the current range of *Papilio homerus* in the eastern and western populations, past populations or capture sites are shown with an X, and *Hernandia* images indicate areas where the host plant is available and could serve as a future site for *P. homerus* release sites if a breeding colony is established. Note the decrease in rainfall that overlaps the eastern population of *P. homerus*.

Changes in precipitation patterns associated with global climate change might also affect the number of remaining *P. homerus*. According to the IPCC Fourth Assessment Report, the Caribbean region will experience increased drought and greater variability in rainfall and drier summers as a result of climate change [58,59,60,61,62]. A decrease in annual rainfall has been documented in the Blue Mountain region, where the eastern *P. homerus* population resides (Figure 6). This decrease in rainfall, coupled with disturbed microclimate patterns associated with deforestation, could potentially decrease the numbers of surviving larvae that require high humidity [6], affecting future population numbers. The increasing intensity of tropical storms due to global climate change will be of greater concern as fragmented areas of tropical forests are vulnerable to extreme weather damage [54]. 

## 8. Invasive Species

It currently is unknown what specific threats invasive species might impose on populations of *P. homerus*. Numerous invasive species now inhabit Jamaica, including plants such as bamboo (*Bambusa vulgaris*), ferns (*Gleichenia* sp. and *Dichranopteris* sp.), and red bead tree (*Adenanthera pavonina*). These invasive plant species could potentially outcompete native *Hernandia* sp., particularly in disturbed areas; however, recent studies have indicated that host plants are common in known *P. homerus* habitats [4,6,7]. Jamaica also hosts invasive species of animals, such as the Antilles coqui (*Eleutherodactylus johnstonei*), marine toad (*Bufo marinus*), and Indian mongoose (*Herpestes auropunctatus*), but their impact on *P. homerus* populations is unknown. In addition, it is not yet known if the parasitoids of *P. homerus* are native or invasive species. Pathogens also pose a threat to rare invertebrates [63] and an invasive pathogenic species could cause a decline in the number of remaining *P. homerus*.

## 9. Knowledge Gaps and Objectives for a Conservation Plan

### 9.1. Determine the Current Geographic Distribution and Estimate the Total Number of Individuals in the Stronghold Populations 

There are currently no accurate estimates of the number of remaining *P. homerus* in either population and the only previous estimates pertain to localized subpopulations [5]. Additional studies using MRR and distance sampling techniques are needed to locate and estimate the number of individuals in other subpopulations within the western and eastern metapopulations, which could be coupled with population viability analyses to determine the probability of population extinctions. We also recommend that future studies use MRR estimators in the program MARK to report estimated population sizes (for open and closed populations) with upper and lower bounds to provide a more comprehensive estimate of the population size.

### 9.2. Determine the Spatial and Temporal Dynamics of P. homerus Subpopulations

It is known that subpopulations experience seasonal fluctuations, but the cause is unknown. In addition, it is unknown if the decrease in number of *P. homerus* individuals in subpopulations is actually a spatial shift in range rather than a temporal change in numbers. Precipitation likely plays a large role; however, determining the other factors that affect subpopulation dynamics could provide information necessary for locating new areas where *P. homerus* could be released. 

### 9.3. Survey Areas to Locate Suitable Habitat for the Potential Release of Captive-Bred P. homerus

Extending the present range of *P. homerus* is a key priority for continued survival. Previous scouting trips have located potential areas that might serve as suitable habitats, including Spanish River Valley and Dolphin Head Mountain, where host plants are abundant (Figure 6) [11]. The areas have high rainfall (annual rainfall of 2500 mm), but they also are susceptible to dry periods, which could prove difficult for the introduction and establishment of *P. homerus*. In addition, reestablishing a central population could act as a corridor to facilitate gene flow between the eastern and western populations.

### 9.4. Regularly Monitor Habitat Quality in Areas where the Current Populations Exist and where Future Populations Could Be Introduced

The abundance of viable host plants, temperature, and seasonal precipitation are important factors that affect *P. homerus* survival and abundance. Data gathered from monitoring the habitat might be useful for establishing new parks or expanding park ranges.

### 9.5. Create Protected Areas of Montane Forest

In order to prevent the clearing of land for agriculture, the homogenization of the landscape, and the creation of forest fragments, there should be an increase in the number of national parks and forests. The establishment of these areas, such as the Blue and John Crow Mountains National Park in 1991, could have *P. homerus* populations located near their center and would provide protection for other endemic and endangered species.

### 9.6. Conduct Research to Determine Ecological Threats to P. homerus and Potential Mitigation Strategies

Ecological threats, such as parasitoids, are responsible for a large percentage of *P. homerus* mortality. It is unknown if the parasitoids that cause high mortality in *P. homerus* (*Ooencyrtus* sp. and *Chrysonotomyta* spp.) use alternate hosts, but if parasitoid populations could be controlled this might increase the abundance of *P. homerus*. Although the impact of parasitoids on larval mortality is documented, there are likely many other factors that need to be identified in order to implement a strategic plan to reduce ecological threats. 

### 9.7. Develop Ex Situ Conservation and Recovery Program

A refugium population for *P. homerus* should be established, followed by captive breeding and reintroductions. A breeding program would include the procurement of wild-collected *P. homerus* to assist in reestablishing stable wild populations. In addition, the cross-breeding of individuals from the western and eastern populations to relieve inbreeding depression would be a priority of a breeding program. Captive-bred individuals could be released as pupae or adults in the eastern and western populations or other regions with suitable habitat. 

### 9.8. Assess Level of Genetic Differentiation between Spatially Discrete Populations

Wing fragments should be sampled from individual *P. homerus* collected from each population, which can be used with PacBio^®^ sequencing technology to discover and develop polymorphic microsatellite markers to determine heterozygosity. In addition, individuals from each population should be crossed to determine if the hybrids are fertile and capable of feeding on different species of *Hernandia* and the relatively widespread *Ocotea* trees. If hybrids are fertile, they can be used to introduce alleles into each population to overcome inbreeding depression.

### 9.9. Educate the Local Public on the Status of the Populations, Conservation Efforts, and the Importance of Keeping These Populations Protected

Continuing to educate people that live near the extant populations of *P. homerus* serves as one of the best lines of defense against illegal poaching. The poaching of *P. homerus* has decreased, likely as a direct result of locals questioning strangers who come into the area.

## 10. Conclusions

Despite efforts to study *P. homerus*, many of the basic principles of its biology continue to be unresolved. Given the long-term interest in *P. homerus*, dating back at least to the 18th century [13], our lack of knowledge is paradoxical. The authors suggest that there are at least two natural circumstances limiting our ability to acquire fundamental information: (1) the difficult terrain of the habitat, and (2) the rarity of *P. homerus*. 

*Papilio homerus* inhabits amphitheater or bowl-like settings [19], common in the Cockpit Country [5] and the Blue and John Crow Mountains [4,6,10], which are problematic for long-term studies. There are few roads that lead to these regions, and traveling the paths (or lack thereof) through the Wet Montane Forests often requires an experienced guide. The lack of field stations also hinders long-term field studies and reduces most observations to day trips at the periphery of the habitat [7]. The Cockpit Country, for instance, is famous for its slippery and sharp rocks, sinkholes, and unexplored caves, which create a setting where one has to move carefully to prevent stepping into a pit or falling into a sinkhole. Although the habitat requirement for *P. homerus* helps protect the species from poaching, which has become less of a concern in recent years [7], it also impedes studies.

*Papilio homerus* is historically rare. An examination of sightings and collections has revealed that *P. homerus* sightings and captures are not necessarily decreasing [7]. The density of this species, therefore, has likely remained low for a long period of time. Species at low densities that inhabit small areas are particularly susceptible to genetic bottlenecking and the continued habitat destruction (small-scale farming and bauxite mining) [9] could have catastrophic effects on the species and raises concern for its extinction. 

Although there is momentum to protect *P. homerus* and its habitat, through workshops and education (involving such groups as the Natural History Society of Jamaica (NHSJ), Portland Environmental Protection Agency (PEPA), the National Environmental Protection Agency (NEPA), and the Cockpit Country Stakeholders Group), and laws put in place to prevent illegal trade (*P. homerus* listed as an endangered species in 1982 and protected by the Third Schedule of the Jamaica Wildlife Protection Act of 1945 and Endangered Species Act of 2000), studies of the genetic diversity should be the highest priority in future investigations. The low density of this species and its shrinking habitat, coupled with the distance between the isolated populations, could result in both populations experiencing a loss of genetic diversity through small population size. Studies of the molecular diversity of the remaining *P. homerus* populations are on the horizon and the results could impact future conservation plans for this distinctive and imperiled butterfly.
